# Pro-oncogenic immunohistochemical markers in Merkel cell carcinoma with or without polyomavirus signature: a Brazilian cohort^؆^

**DOI:** 10.1016/j.abd.2026.501438

**Published:** 2026-09-01

**Authors:** Aline Siqueira Talarico, Murilo de Oliveira Lima Carapeba, Claudio de Lelis Filgueiras de Souza, Alexandre Ozores Michalany, Thiago Jeunon de Sousa Vargas, Gustavo Gonçalves Engelman, Rute Facchini Lellis, Rafael Fantelli Stelini, Julio Cesar Moraes, Fernanda Caramella Pereira, Isabel Cristina Gomes Moura, Daniela Cristina Gonçalves, Adilson Da Costa

**Affiliations:** a Department of Postgraduate Program in Health Science, Instituto de Assistência Médica ao Servidor Público Estadual, São Paulo, SP, Brazil; b Department of Dermatology, Universidade do Oeste Paulista, Presidente Prudente, SP, Brazil; c Department of Adult Health, Universidade de Alfenas, Alfenas, MG, Brazil; d Laboratório Paulista de Dermatopatologia, São Paulo, SP, Brazil; e Laboratório Investigação em Dermatologia, Rio de Janeiro, RJ, Brazil; f Centro de Patologia de Pouso Alegre, Pouso Alegre, MG, Brazil; g Department of Pathology, Faculty of Medical Sciences, Santa Casa de São Paulo, São Paulo, SP, Brazil; h Department of Pathology, Universidade Estadual de Campinas, São Paulo, Brazil; i Imunocel Laboratorial, São Paulo, SP, Brazil; j Department of Pathology, School of Medicine, Johns Hopkins University, Baltimore, MD, United States of America; k Department of Statistics, Universidade Federal de Minas Gerais, Belo Horizonte, MG, Brazil; l Department of Classical and Vernacular Languages, Universidade de São Paulo, São Paulo, SP, Brazil; m Department of Laboratory Medicine and Pathology, Mayo Clinic, Rochester, MN, United States of America

Dear Editor,

Merkel Cell Carcinoma (MCC) is a rare cutaneous neuroendocrine neoplasm with a high mortality rate. Its pathogenesis correlates with the presence of Merkel Cell Polyomavirus (MCPyV) or Ultraviolet Radiation (UV) exposure, and the relative contribution of each pathway may vary by geographic region according to local UV radiation intensity.^1^

The objective of this study was to assess MCPyV expression in a Brazilian MCC cohort and to correlate its presence with pro-oncogenic Immunohistochemical (IHC) markers (IRB approval number: 5.579.442). We conducted a retrospective study of 27 MCC cases diagnosed between 2014 and 2021 at a reference center. Clinical data, including gender and anatomical site, were reviewed, and IHC staining was performed to evaluate MCPyV, p53, TNF-α, GLI1, SHH, PKCε, PD-L2, IFN-γ, RhoA, RhoC, STAT3, and AKT-1 expression, based on emerging evidence of their relevance in MCC tumor biology. All stains were independently evaluated by two dermatopathologists; cases with interpretative discrepancies were reviewed by two dermatopathology-trained dermatologists. Qualitative variables are reported as counts and percentages, and quantitative data are presented as minimum, maximum, mean, standard deviation (SD), median, and first (Q1) and third quartiles (Q3). No variables in the dataset contained missing values.

Table 1 shows that most patients were women (55.6%) with lesions predominantly located in sun-exposed skin, especially the head and neck (74.1%), and this pattern did not vary according to MCPyV status. The levels of immunohistochemical marker expression are also presented in Table 1 and are observed in Figs. 1 to 3. MCPyV positivity was observed in 63% of cases. Even though one might expect a higher proportion of virus-negative tumors in Brazil, given high UV-exposure and elevated rates of skin cancer, our findings are consistent with the first report examining MCPyV prevalence in Brazilian MCC patients, which documented positivity in about 60% of cases.^1^, ^2^

**Table 1:** Sample characterization.

Variable	n (%)
Gender	
F (female)	15 (55.6%)
M (male)	12 (44.4%)
Site of tumor	
Non sun-exposed	7 (25.9%)
Sun exposed	20 (74.1%)
Markers positivity	
MCPyV (Merkel Cell Polyomavirus)	17 (63.0%)
GLI-1 (Glioma-associated homologue-1)	0 (-)
SHH (Sonic hedgehog protein)	0 (-)
RhoA (Ras homolog family member A)	25 (92.6%)
RhoC (Ras homolog family member C)	27 (100.0%)
Stat-3 (signal transducer and activator of transcription 3)	27 (100.0%)
TNF-α (tumor necrosis factor-alpha)	11 (40.7%)
IFN-γ (interferon gamma)	22 (81.5%)
AkT-1 (serine/threonine kinase AKT)	27 (100.0%)
p53 (protein 53)	5 (18.5%)
PKCε (protein kinase C epsilon)	23 (85.2%)
PD-L2 (programmed death-ligand 1)	25 (92.6%)

**Fig. 1:**
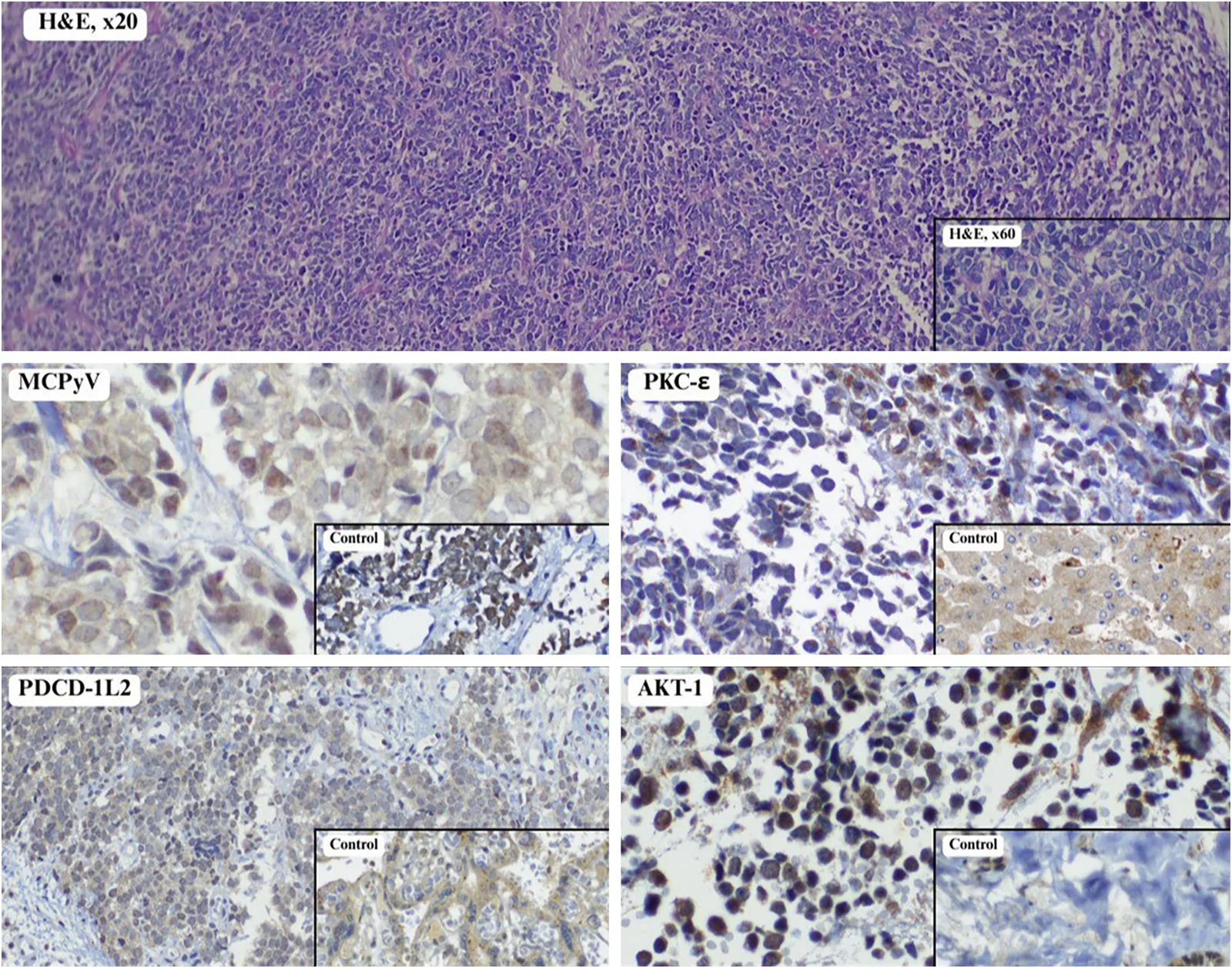
Immunohistochemical evaluation of MCPyV, PKCε, PD-L2, and AKT-1 expression. Representative immunohistochemical staining in Merkel Cell Carcinoma (MCC), including Hematoxylin-Eosin (H&E) morphology and expression of MCPyV, PKCε, PD-L2, and AKT-1. MCPyV positivity was observed in approximately 63% of cases, showing predominantly nuclear staining in tumor cells. PKCε exhibited cytoplasmic positivity, with significantly higher expression in MCPyV-positive tumors. PD-L2 demonstrated strong membranous and cytoplasmic staining in 92.6% (25/27) of cases, with high proportions of marked tumor cells, supporting immune checkpoint activation. AKT-1 showed diffuse cytoplasmic positivity in 100% of tumors, with moderate-to-strong intensity, indicating activation of the PI3K/AKT pathway.

**Fig. 2:**
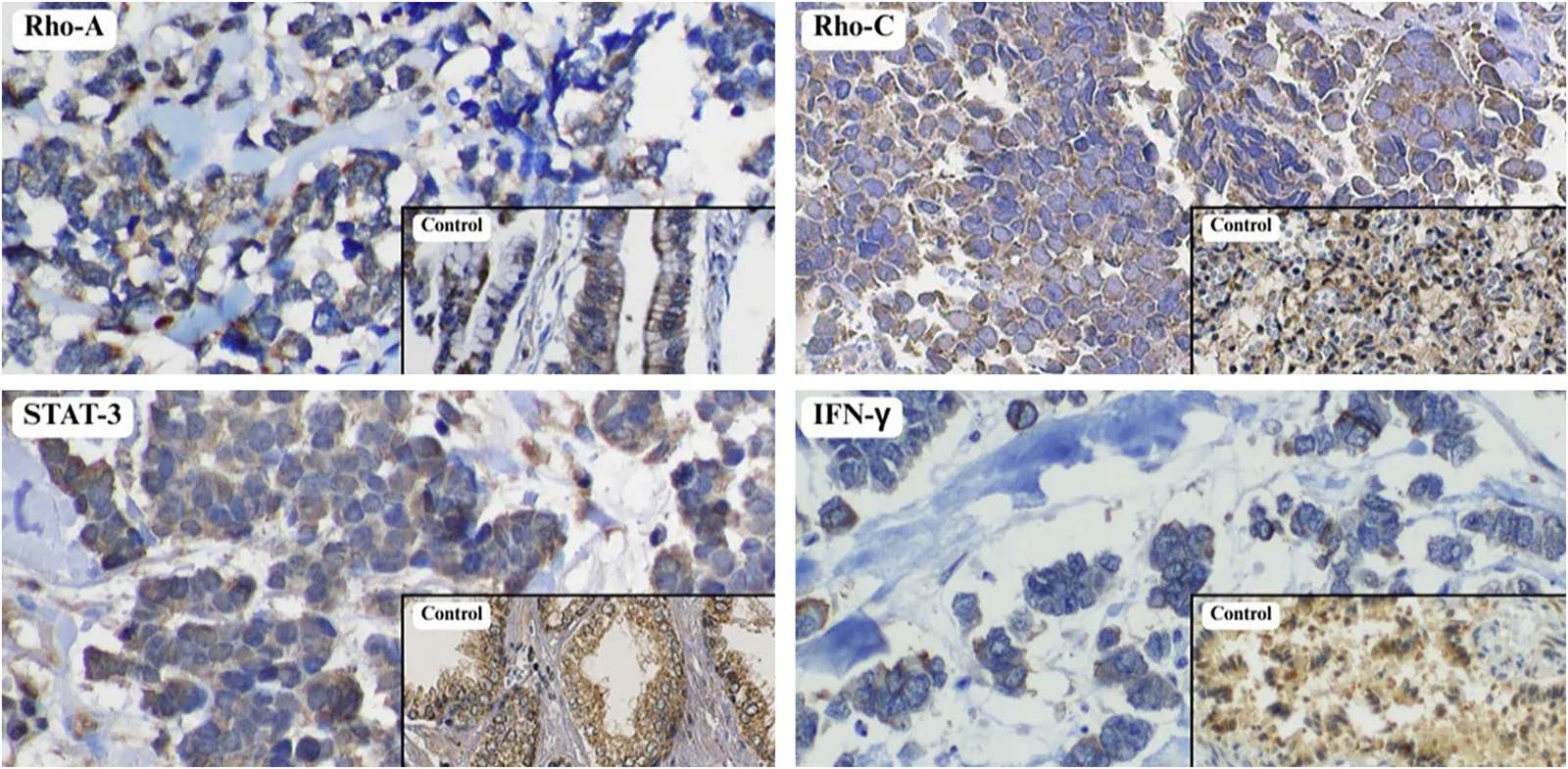
Immunohistochemical characterization of RhoA, RhoC, STAT3, and IFN-γ expression highlighting Rho/STAT pathway activation. Immunohistochemical expression of RhoA, RhoC, STAT3, and IFN-γ in MCC samples. RhoC and STAT3 showed diffuse and strong positivity in 100% of cases, with cytoplasmic staining for RhoC and predominantly nuclear staining for STAT3, involving a high percentage of tumor cells. RhoA demonstrated cytoplasmic positivity in 92.6% of tumors, with higher expression in MCPyV-positive and sun-exposed lesions. IFN-γ expression was low or absent in most cases, showing weak and focal cytoplasmic staining in a small proportion of tumor cells, indicating limited immune activation.

**Fig. 3:**
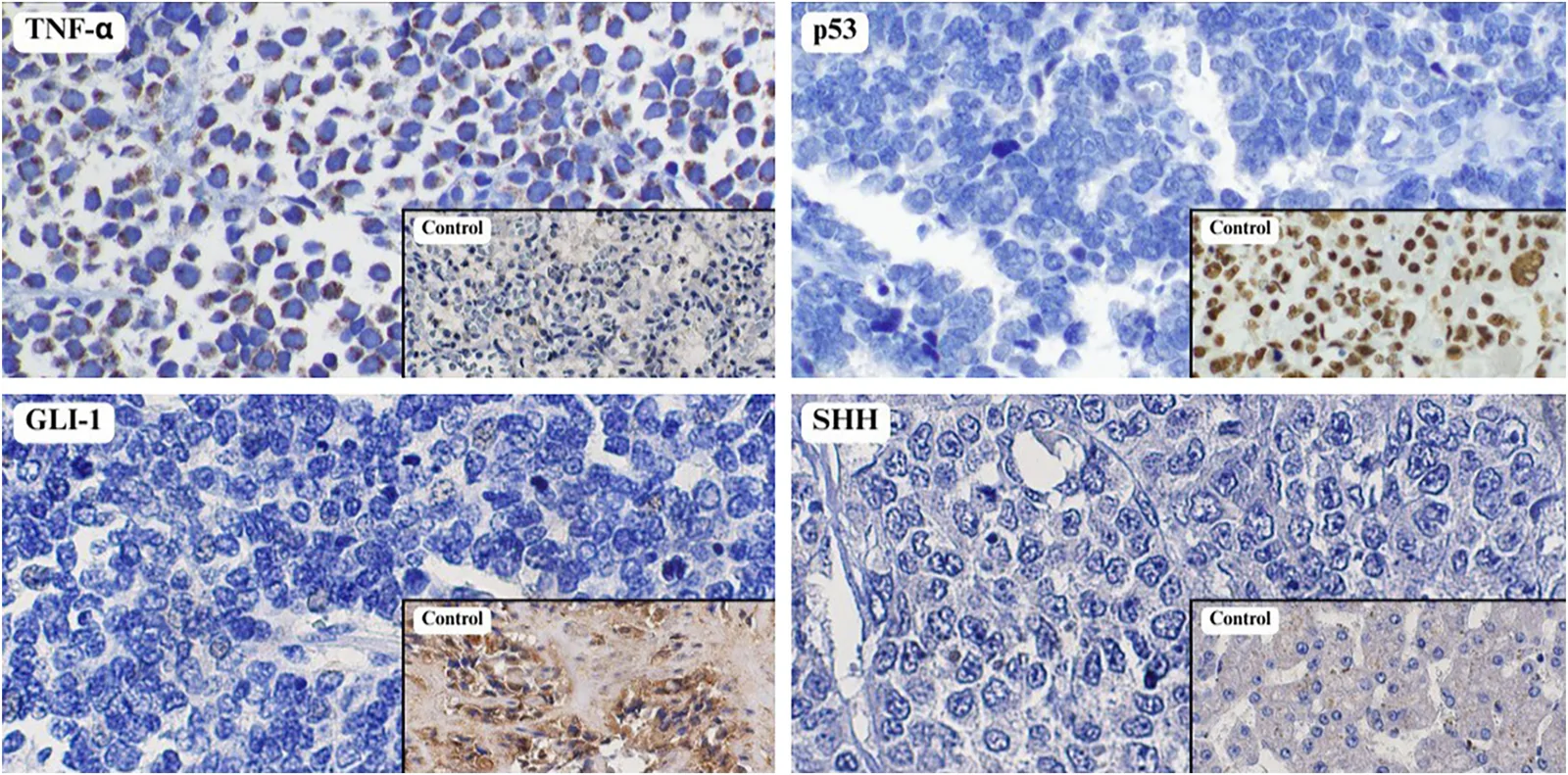
Immunohistochemical assessment of TNF-α, p53, GLI-1, and SHH expression demonstrating limited tumor suppressor and Hedgehog pathway activation. Legend: Immunohistochemical analysis of TNF-α, p53, GLI-1, and SHH in MCC. TNF-α showed low-to-moderate cytoplasmic positivity, without significant differences according to MCPyV status. p53 expression was generally low, with weak nuclear staining in a small percentage of tumor cells. GLI-1 and SHH were negative in all cases, with absence of nuclear (GLI-1) and cytoplasmic (SHH) staining, indicating no detectable activation of the Hedgehog pathway in this cohort.

Table 2 shows that RhoC, STAT3, and AKT-1 were positive in 100% of tumors, while RhoA and PD-L2 were positive in 92.6%. PKCε was significantly higher in MCPyV-positive tumors (3.1 vs. 1.0; p < 0.001). RhoA (p = 0.003) and AKT-1 (p = 0.018) were also elevated in MCPyV-positive cases. Additionally, Table 3 shows that RhoA was higher in sun-exposed tumors (p = 0.013). TNF-α and p53 expressions remained modest, and IFN-γ expression was low in both groups.

**Table 2:** Markers mean according to MCPyV positivity.

Markers	MCPyV negative	MCPyV positive
GLI-1	0 (−)	0 (−)
SHH	0 (−)	0 (−)
RhoA (p = 0.003)	2.3 (1.1)	3.5 (0.6)
RhoC (p = 0.451)	3.3 (0.7)	3.5 (0.7)
Stat-3 (p = 0.118)	3.0 (0.7)	3.4 (0.6)
TNF-α (p = 0.210)	0.3 (0.5)	0.8 (0.9)
IFN-γ (p = 0.279)	1.6 (1.3)	2.2 (1.3)
AkT-1 (p = 0.018)	2.3 (1.3)	3.4 (0.8)
p53 (p = 0.437)	0.3 (0.9)	0.5 (1.1)
PKCε (p < 0.001)	1.0 (1.1)	3.1 (1.0)
PD-L2 (p = 0.252)	2.1 (1.7)	2.8 (1.3)

Data presented as mean (dp). P values refer to the Wilcoxon test.

**Table 3:** Markers mean according to site of tumor.

Markers	Non exposed	Exposed
GLI-1	0 (−)	0 (−)
SHH	0 (−)	0 (−)
RhoA (p = 0.013)	2.4 (0.5)	3.2 (1.0)
RhoC (p = 0.580)	3.3 (0.8)	3.4 (0.7)
Stat-3 (p = 0.197)	3.0 (0.6)	3.4 (0.7)
TNF-α (p = 0.084)	0.1 (0.4)	0.8 (0.9)
IFN-γ (p = 0.100)	1.3 (1.0)	2.2 (1.4)
AkT-1 (p = 0.089)	2.4 (1.1)	3.2 (1,1)
p53 (p = 0.153)	0 (−)	0.6 (1.1)
PKCε (p = 0.246)	1.7 (1.7)	2.5 (1.3)
PD-L2 (p = 0.152)	1.9 (1.6)	2.8 (1.4)

Data presented as mean (dp). P values refer to the Wilcoxon test.

Despite the high level of UV exposure in this population and the predominance of lesions in sun-exposed skin, p53 expression remained low regardless of MCPyV status, supporting previous observations.^3^

TNF-α is a pro-inflammatory cytokine involved in multiple neoplasms, with the capacity to promote both tumor proliferation and antitumor immune responses.^4^ In MCC, our cohort showed that TNF-α positivity did not show statistically significant associations with the other markers, preventing definitive conclusions regarding its role.

GLI1 and SHH have been proposed as indicators of Hedgehog (Hh) pathway activation in MCPyV-positive MCC, supported by evidence linking Hh pathway activation to virus-mediated oncogenesis.^5^ However, contrary to those findings, GLI1 and SHH expression were absent in all cases in our cohort, regardless of MCPyV status. This variability across different populations highlights the need for additional research into the Hh pathway in MCC.

Within the signaling pathways and markers of MCC oncogenesis, our data regarding AkT-1 pathway positivity support the findings already published by Iwasaki et al. as one of the main pathways described in MCC oncogenesis.^6^ However, the overall positivity found in our study is higher than that described, with all cases showing high levels of expression.

Phosphorylated PKCε has a strong association with MCPyV positivity, a fact that motivated the testing of PKCε in this study.^7^ In the absence of previous studies, we observed high positivity in exposed areas, as well as in covered areas in the polyoma-positive group, reinforcing the importance of this pathway in MCC-VP (Merkel-cell carcinoma virus-positive).

Both PD-1 and its ligands are closely related to the characteristic immune escape of MCC, and recent immune checkpoint therapies used in MCC are directed at anti-PD-1 and/or anti-PD-L1, which has significantly changed the therapeutic landscape of the disease.^8^ These data led us to study PD1-L2, which showed positivity in 25 of 27 cases (92.6%), regardless of the polyomavirus status, with a high median score of positivity of 3. This is an unprecedented datum in research with MCC, despite which etiological subtype it might be.

The Rho pathway, already reported in MCC, RhoA only associated with the MCC-VP, has shown a correlation with an aggressive migratory phenotype.^9^ Its correlation with MCC-VN (Merkel-cell carcinoma virus-negative) is unprecedented, as is the RhoC pathway, with our data being positive in all cases, regardless of viral status.

Further studies are still needed to better understand the complex Rho-STAT relationship, but it is known that Rho GTPases can act as regulators of the non-canonical STAT signaling pathway, contributing to the proliferative capacity and invasion of various types of cancers,^10^ a relationship not yet described in MCC. The present study demonstrates the importance of STAT3 as well as RhoA and RhoC in MCC. Although analyzed independently, these oncogenic pathways may be interconnected, allowing new studies to clarify their relationships and also promote new immune checkpoint therapies.

Although our findings provide new and meaningful data to the medical literature, this study is limited by the relatively small sample size in an already-considered rare neoplasia. Larger multicenter studies will be necessary to better clarify the associations presented here. Nevertheless, the data reported in this work establish an important reference point for future research and encourage the exploration of additional intermediate or interconnected pathways involved with the markers that we studied, which are extremely helpful to better elucidate the pathogenesis of MCC and potential upcoming therapeutic strategies.

In conclusion, PD-L2 positivity reinforces the immune escape characteristic of MCC. PKCε, RhoA, and AKT prominence in MCPyV-positive cases suggests therapeutic relevance, with RhoA also consistently elevated in sun-driven tumors. RhoA and PKCε may represent distinct targetable pathways, respectively linked to sun-damage and viral signaling. The novel biomolecular pathways identified in this study are summarized in Fig. 4.

**Fig. 4:**
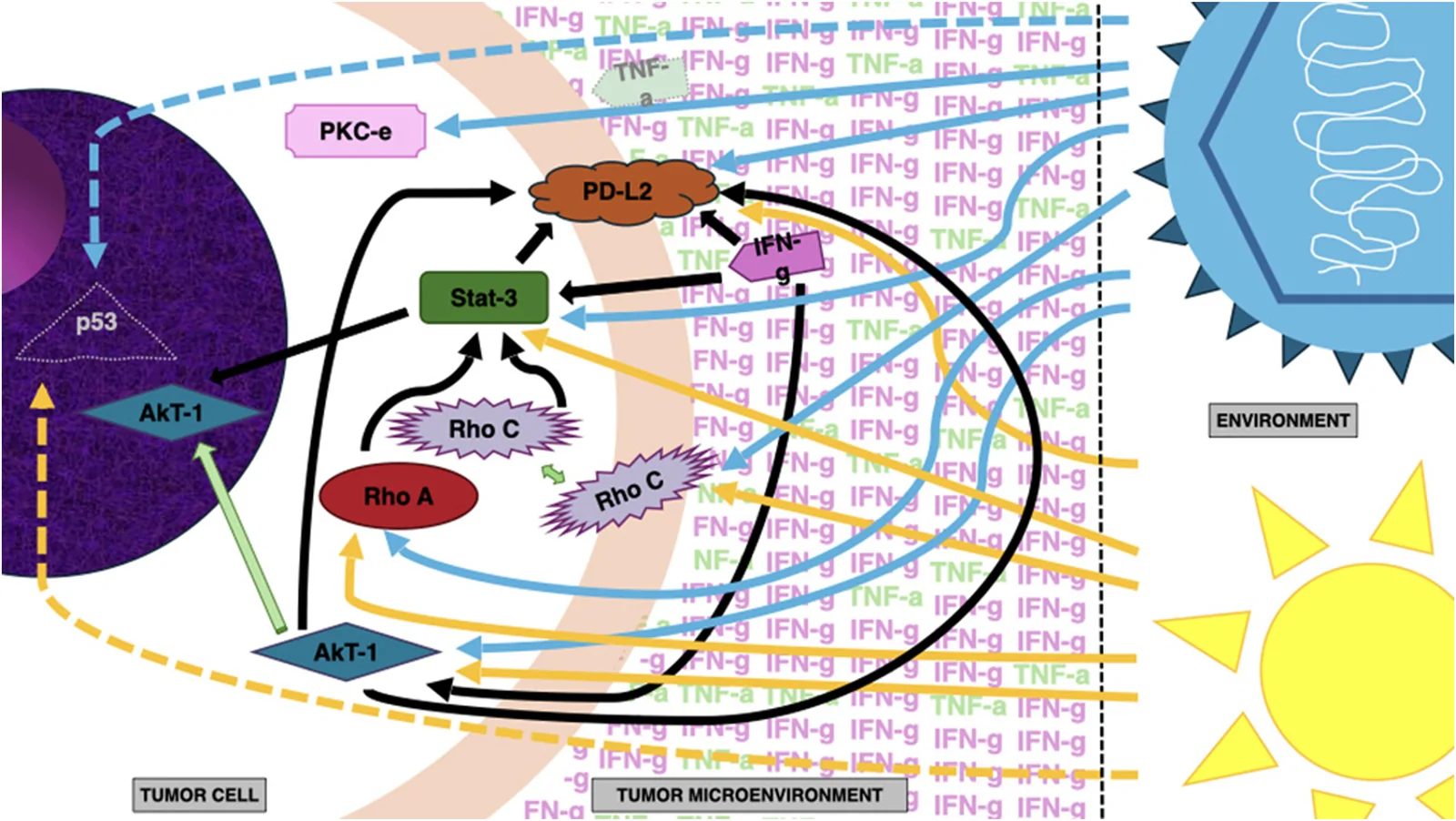
The biomolecular pathways presented in this study. Summary of the main tumor signaling pathways of Merkel cell carcinoma evaluated in the present study and their observed interconnections. MCPyV, Merkel Cell Polyomavirus; GLI-1, Glioma-associated homologue-1; SHH, Sonic hedgehog protein; RhoA, Ras homolog family member A; RhoC, Ras homolog family member C; Stat-3, Signal transducer and activator of transcription-3); TNF-α, Tumor Necrosis Factor-alpha; IFN-γ, Interferon gamma); AkT-1, Serine/Threonine kinase AKT; p53, Protein 53; PKCε, Protein Kinase C epsilon; PD-L2, Programmed death-ligand-1.

## ORCID ID

Aline Siqueira Talarico: 0009-0004-7788-4729

Murilo de Oliveira Lima Carapeba: 0000-0002-0817-932X

Claudio de Lelis Filgueiras de Souza: 0000-0002-5684-7222

Alexandre Ozores Michalany: 0000-0002-8814-4513

Thiago Jeunon de Sousa Vargas: 0000-0003-1750-438X

Gustavo Gonçalves Engelman: 0009-0007-1248-4996

Rute Facchini Lellis: 0000-0001-7690-0513

Rafael Fantelli Stelini: 0000-0003-0618-1693

Julio Cesar Moraes: 0009-0006-4296-6550

Fernanda Caramella Pereira: 0009-0001-4087-3186

Isabel Cristina Gomes Moura: 0000-0002-5549-3426

Daniela Cristina Gonçalves: 0009-0002-0108-6486

## Financial support

This study received partial financial support through fellowship resources from the Fundo de Apoio à Dermatologia (FUNADERM), Brazil.

## Authors’ contributions

Aline Siqueira Talarico: The conception and design of the study; data collection, or data analysis and interpretation; statistical analysis; writing of the article or critical revision of important intellectual content; data acquisition, analysis, and interpretation; critical review of the literature.

Murilo de Oliveira Lima Carapeba: Data collection, or data analysis and interpretation; data acquisition, analysis, and interpretation.

Claudio L. F. Souza: Data collection, or data analysis and interpretation.

Alexandre Ozores Michalany: Data collection, or data analysis and interpretation.

Thiago Jeunon de Sousa Vargas: Data collection, or data analysis and interpretation.

Gustavo Gonçalves Engelman: Data collection, or data analysis and interpretation.

Rute Facchini Lellis: Data collection, or data analysis and interpretation.

Rafael Fantelli Stelini: Data collection, or data analysis and interpretation.

Julio Cesar Moraes: Data collection, or data analysis and interpretation.

Fernanda Caramella Pereira: Intellectual involvement in the diagnostic and/or therapeutic management of the studied cases; critical review of the literature.

Isabel Cristina Gomes Moura: Formal analysis.

Daniela Cristina Gonçalves: Critical review of the literature.

Adilson Da Costa: The conception and design of the study; active participation in guiding the research; intellectual involvement in the diagnostic and/or therapeutic management of the studied cases; critical review of the literature; final approval of the final version of the manuscript.

## Research data availability

The entire dataset supporting the results of this study was published in this article.

## Conflicts of interest

None declared.

## References

[1] Siqueira S.O.M., Campos-do-Carmo G., Santos A.L.S., Martins C., Melo A.C. (2023). Merkel cell carcinoma: epidemiology, clinical features, diagnosis and treatment of a rare disease. An Bras Dermatol..

[2] Neto C.F., Oliveira W.R.P., Costa P.V.A., Cardoso M.K., Barreto P.G., Romano C.M. (2019). The first observation of the association of Merkel cell polyomavirus and Merkel cell carcinoma in Brazil. Int J Dermatol..

[3] Öğüt B., Bayram E.K., İnan M.A., Kestel S., Erdem Ö. (2023). Association of Merkel cell polyomavirus status with p53, RB1, and PD-L1 expression and patient prognosis in Merkel cell carcinomas: clinical, morphologic, and immunohistochemical evaluation of 17 cases. Appl Immunohistochem Mol Morphol.

[4] Temur B.Z., Timucin A.C., Atik A.E., Kocagoz T., Can O. (2025). Peptide-based regulation of TNF-α-mediated cytotoxicity. Biomolecules..

[5] Kuromi T., Matsushita M., Iwasaki T., Nonaka D., Kuwamoto S., Nagata K. (2017). Association of expression of the hedgehog signal with Merkel cell polyomavirus infection and prognosis of Merkel cell carcinoma. Hum Pathol..

[6] Iwasaki T., Matsushita M., Nonaka D., Kuwamoto S., Kato M., Murakami I. (2015). Comparison of Akt/mTOR/4E-BP1 pathway signal activation and mutations of PIK3CA in Merkel cell polyomavirus-positive and -negative carcinomas. Hum Pathol..

[7] Costa A., Mackelfresh J., Gilbert L., Bonner M.Y., Arbiser J.L. (2017). Activation of protein kinase C epsilon in Merkel cell polyomavirus-induced Merkel cell carcinoma. JAMA Dermatol..

[8] Stachyra K., Dudzisz-Śledź M., Bylina E., Szumera-Ciećkiewicz A., Spałek M.J., Bartnik E. (2021). Merkel cell carcinoma: from molecular pathology to novel therapies. Int J Mol Sci..

[9] Stakaitytė G., Nwogu N., Dobson S.J., Knight L.M., Wasson C.W., Salguero F.J. (2018). Merkel cell polyomavirus small T antigen drives cell motility via Rho-GTPase-induced filopodium formation. J Virol..

[10] Corry J., Mott H.R., Owen D. (2020). Activation of STAT transcription factors by the Rho-family GTPases. Biochem Soc Trans..

